# Correction: Iinuma et al. Utility of Neutrophil-to-Lymphocyte Ratio, Platelet-to-Lymphocyte Ratio, and Systemic Immune Inflammation Index as Prognostic, Predictive Biomarkers in Patients with Metastatic Renal Cell Carcinoma Treated with Nivolumab and Ipilimumab. *J. Clin. Med.* 2021, *10*, 5325

**DOI:** 10.3390/jcm15176780

**Published:** 2026-09-01

**Authors:** Koji Iinuma, Torai Enomoto, Kei Kawada, Shota Fujimoto, Takashi Ishida, Kimiaki Takagi, Shingo Nagai, Hiroki Ito, Makoto Kawase, Chie Nakai, Kota Kawase, Daiki Kato, Manabu Takai, Keita Nakane, Koji Kameyama, Takuya Koie

**Affiliations:** 1 Department of Urology, Gifu University Graduate School of Medicine, Gifu 5011194, Japan; kiinuma@gifu-u.ac.jp (K.I.); buki2121@gifu-u.ac.jp (M.K.); chie_na@gifu-u.ac.jp (C.N.); stnf55@gifu-u.ac.jp (K.K.); andreas7@gifu-u.ac.jp (D.K.); takai_mb@gifu-u.ac.jp (M.T.); keitaco@gifu-u.ac.jp (K.N.); 2 Department of Urology, Matsunami General Hospital, Hashima-gun 5016062, Japan; try@mghg.jp; 3 Department of Urology, Gifu Prefectural General Medical Center, Gifu 5008717, Japan; keinedvedon@yahoo.co.jp; 4 Department of Urology, Ogaki Municipal Hospital, Ogaki 5038502, Japan; uro2@omh.ogaki.gifu.jp; 5 Department of Urology, Gifu Municipal Hospital, Gifu 5008513, Japan; justaskaxis@gmail.com; 6 Department of Urology, Daiyukai Daiichi Hospital, Ichinomiya 4918551, Japan; kimiaki_takagi5619@yahoo.co.jp; 7 Department of Urology, Toyota Memorial Hospital, Toyota 4718513, Japan; shingo-nagai@nifty.com (S.N.); seanoel2@gmail.com (H.I.); 8 Department of Urology, Kizawa Memorial Hospital, Minokamo 5058503, Japan; i2001029@yahoo.co.jp

**Error in Figure**

In the original publication [1], there was a mistake in the published version of Figure 2. The number of patients at risk in the subgroup analyses according to the neutrophil-to-lymphocyte ratio, platelet-to-lymphocyte ratio, and systemic immune inflammation index was incorrect.

The corrected Figure 2 appears below. The authors state that the scientific conclusions are unaffected. This correction was approved by the Academic Editor. The original publication has also been updated.

## Figures and Tables

**Figure 2:**
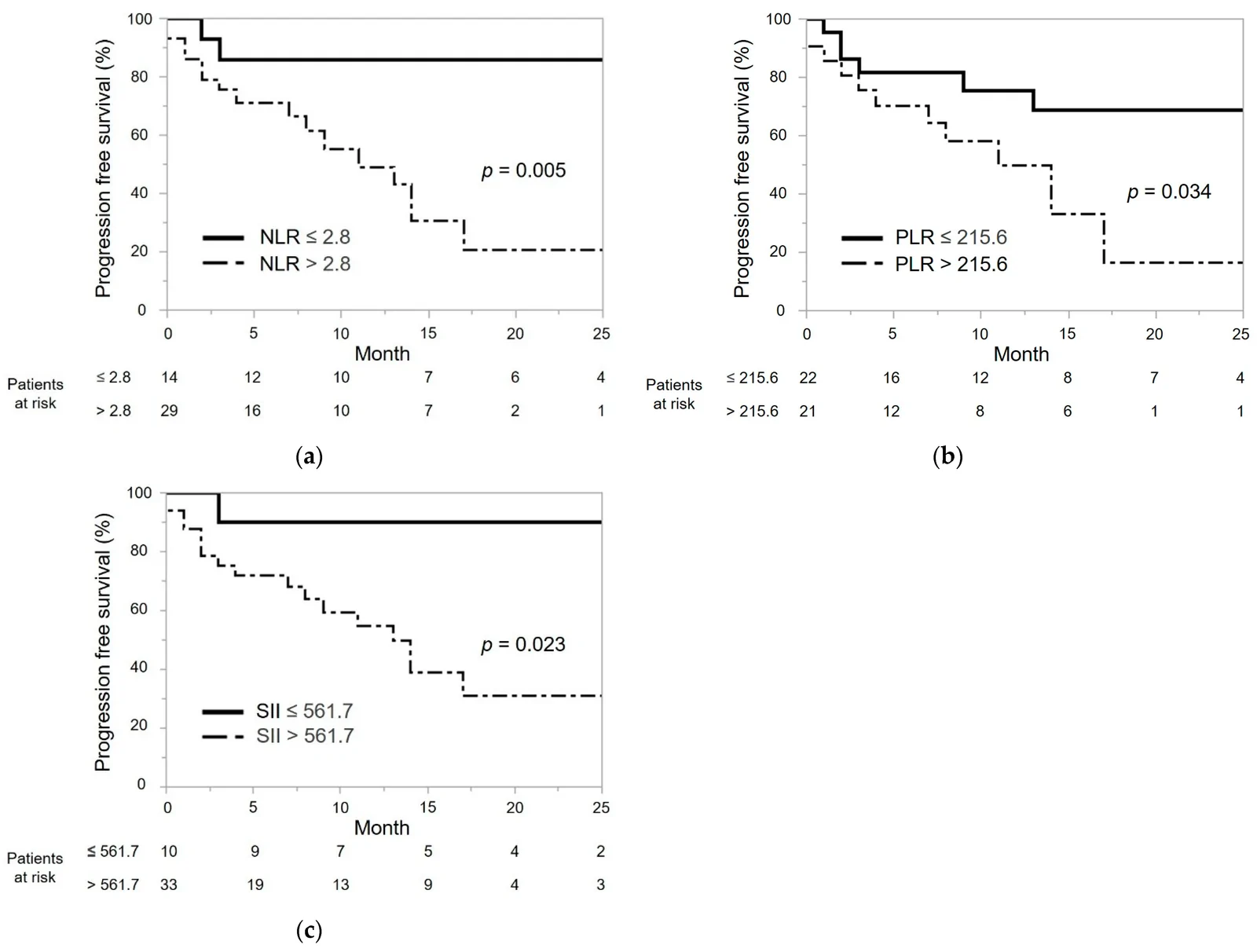
Kaplan–Meier curves illustrating progression-free survival (PFS): (**a**) per neutrophil-to-lymphocyte ratio (NLR) with a cutoff value of 2.8; (**b**) per platelet-to-lymphocyte ratio (PLR) with a cutoff value of 215.6; (**c**) per systemic immune inflammation index (SII) with a cutoff value of 561.7. The 1-year PFS rates were 85.7% and 49.1% for NLR ≤ 2.8 and >2.8, respectively (*p* = 0.005; (**a**)), and 75.5% and 49.7% for PLR ≤ 215.6 and >215.6, respectively (*p* = 0.034; (**b**)). Regarding SII, the 1-year PFS rates were 90.0% and 54.8% when SII was ≤561.7 and >561.7, respectively (*p* = 0.023; (**c**)).
